# Regulation of BMAL1 Protein Stability and Circadian Function by GSK3β-Mediated Phosphorylation

**DOI:** 10.1371/journal.pone.0008561

**Published:** 2010-01-01

**Authors:** Saurabh Sahar, Loredana Zocchi, Chisato Kinoshita, Emiliana Borrelli, Paolo Sassone-Corsi

**Affiliations:** 1 Department of Pharmacology, University of California Irvine, Irvine, California, United States of America; 2 Department of Microbiology and Molecular Genetics, University of California Irvine, Irvine, California, United States of America; 3 Unite 904 INSERM ‘Epigenetics and Neuronal Plasticity’, University of California Irvine, Irvine, California, United States of America; Roswell Park Cancer Institute, United States of America

## Abstract

**Background:**

Circadian rhythms govern a large array of physiological and metabolic functions. To achieve plasticity in circadian regulation, proteins constituting the molecular clock machinery undergo various post-translational modifications (PTMs), which influence their activity and intracellular localization. The core clock protein BMAL1 undergoes several PTMs. Here we report that the Akt-GSK3β signaling pathway regulates BMAL1 protein stability and activity.

**Principal Findings:**

GSK3β phosphorylates BMAL1 specifically on Ser 17 and Thr 21 and primes it for ubiquitylation. In the absence of GSK3β-mediated phosphorylation, BMAL1 becomes stabilized and BMAL1 dependent circadian gene expression is dampened. Dopamine D2 receptor mediated signaling, known to control the Akt-GSK3β pathway, influences BMAL1 stability and *in vivo* circadian gene expression in striatal neurons.

**Conclusions:**

These findings uncover a previously unknown mechanism of circadian clock control. The GSK3β kinase phosphorylates BMAL1, an event that controls the stability of the protein and the amplitude of circadian oscillation. BMAL1 phosphorylation appears to be an important regulatory step in maintaining the robustness of the circadian clock.

## Introduction

Circadian (from the Latin *circa diem* meaning “about a day”) rhythms occur with a periodicity of about 24 hours and enable organisms to adapt and anticipate environmental changes. Circadian control provides an evolutionary advantage to organisms in adapting their behavior and physiology to the appropriate time of day [1], [2]. Feeding behavior, sleep-wake cycles, hormonal levels and body temperature are just a few examples of physiological circadian rhythms.

From a molecular standpoint, circadian rhythms are regulated by transcriptional and post-translational feedback loops generated by a set of interplaying clock proteins. The positive limb of the mammalian clock machinery is comprised of CLOCK and BMAL1, which are transcription factors that heterodimerize through the PAS domain and induce the expression of clock-controlled genes by binding to their promoters at E-boxes. Cryptochromes (*Cry 1*, *Cry2*) and Period genes (*Per1*, *Per2*, *Per3*) are clock-controlled genes that encode proteins that form the negative limb of the circadian machinery. PER and CRY proteins are classically thought to translocate into the nucleus to inhibit CLOCK∶BMAL1 mediated transcription, thereby closing the negative feedback loop [1].

Various core clock proteins undergo post-translational modifications (PTMs), a feature that is likely to contribute significantly to the plasticity of the circadian system. PTMs have been shown to regulate distinct functions, including transcriptional activation and intracellular localization. PTMs have been proposed to participate in controlling the timing between the activation and the repression of circadian transcription [3]. Among the clock proteins, BMAL1 undergoes an extensive repertoire of PTMs, including phosphorylation [4], [5], [6], acetylation [7], sumoylation [8], [9] and ubiquitylation [10]. Yet, the signaling pathways controlling the stability of the BMAL1 protein have not been deciphered. In the present study we have identified GSK3β as a critical regulator of BMAL1 stability and activity. GSK3β is a ubiquitous kinase which regulates various cellular functions, ranging from glucose homeostasis, to cell survival and cell-fate specification [11]. GSK3β has been linked to various pathological conditions such as diabetes, Alzheimer's, cancer and bipolar disorder [12], [13], [14], [15].

The role of GSK3β in circadian control has been reported. The first evidence was based on the observation that *shaggy* (*sgg*), the *Drosophila* ortholog of GSK3, controls the period of circadian locomotor activity by phosphorylating TIMELESS and regulating nuclear translocation of the PERIOD/TIMELESS heterodimer [16], [17]. Recently a high-throughput approach demonstrated that inhibition of GSK3β leads to shortening of the period in cultured mammalian cells [18]. In mammals, GSK3β has been reported to phosphorylate PER2, CRY2 and Rev-erbα. It has also been reported that the kinase activity of GSK3β oscillates in the central mammalian clock, suprachiasmatic nucleus (SCN) and in the peripheral clocks (liver and fibroblasts) [19]. GSK3β mediated phosphorylation appears to have differential effect on the stability of the targeted substrates, since it has been shown to induce the degradation of CRY2 [20] and the stabilization of Rev-erbα [21]. Here we show that GSK3β specifically phosphorylates BMAL1 and primes it for ubiquitylation, followed by proteasomal degradation. This control mechanism significantly influences the efficacy and amplitude of circadian gene expression.

## Results

### GSK3β Phosphorylates BMAL1 and Primes It for Ubiquitylation

**Figure 1:**
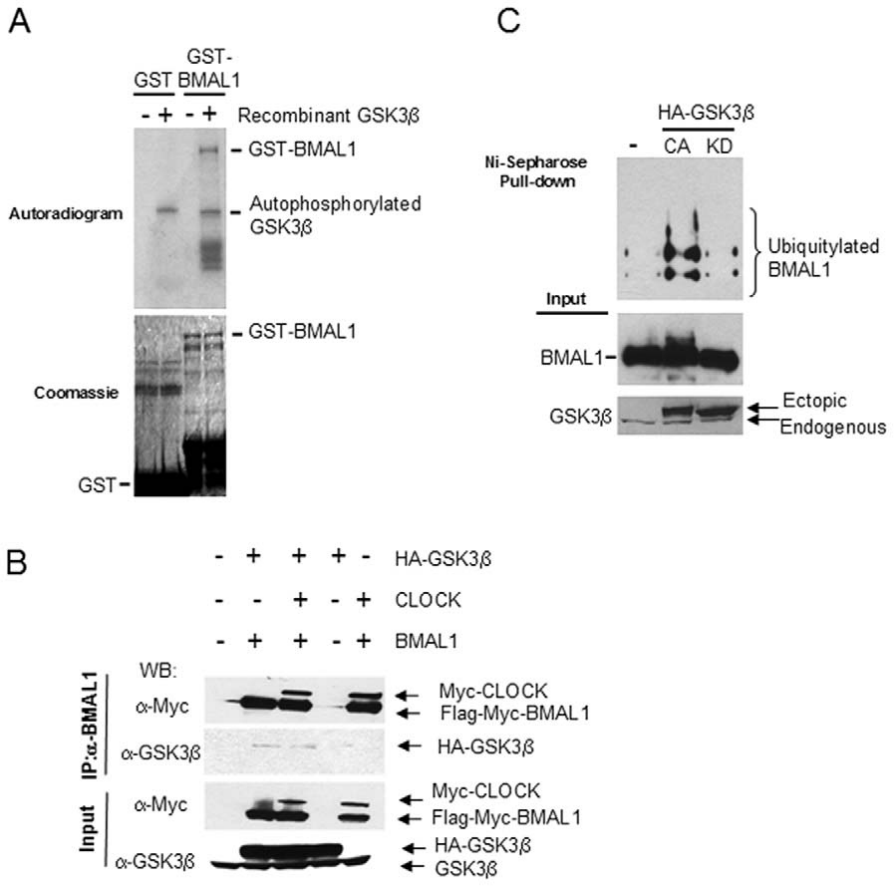
GSK3β phosphorylates BMAL1 and primes it for ubiquitylation. (A) *In vitro* Kinase assay. Bacterially purified GST and GST-BMAL1 were incubated with recombinant GSK3β in presence of γ^32^P-ATP. Top panel shows the autoradiogram; bottom panel shows the coomassie blue staining for the same gel. (B) HEK 293 cells were transfected with plasmids expressing Flag-myc-BMAL1, myc-CLOCK with or without HA-GSK3β. Total lysates were prepared and subjected to immunoprecipitation using anti-BMAL1 antibody. Immunoprecipitated proteins were detected by Western blotting (WB) using indicated antibodies. (C) Ubiquitylation assay. HEK 293 cells were transfected with plasmids expressing HIS-Ubiquitin, Flag-myc-BMAL1, myc-CLOCK, with or without the constitutively active (CA) or kinase dead (KD) mutants of HA- tagged GSK3β. Ubiquitylated BMAL1 was detected by Western analysis using anti-Flag antibody. Input samples were probed with anti-Flag (to detect BMAL1) and anti-GSK3β antibodies.

BMAL1 is a phosphoprotein targeted by various kinases [4], [5], [6]. We noted that BMAL1 contains 15 sites with the consensus T/SXXXS/T phosphoacceptor sequence for GSK3β. This prompted us to determine whether GSK3β can phosphorylate BMAL1. We performed *in vitro* kinase assays on bacterially purified GST-BMAL1 or GST alone by incubating them with recombinant GSK3β in presence of γ^32^P-ATP. Our results demonstrate that GST-BMAL1 was readily phosphorylated by GSK3β, whereas GST alone, although expressed at much greater levels, was not phosphorylated under equivalent conditions (Fig. 1A). Thus, BMAL1 appears to be an efficient substrate of GSK3β. Furthermore, to detect physical association between BMAL1 and GSK3β, HEK 293 cells were transiently transfected with plasmids expressing BMAL1 and CLOCK in the presence or absence of GSK3β. Immunoprecipitation of BMAL1, either in presence or absence of CLOCK, pulled down GSK3β, confirming that BMAL1 and GSK3β do interact physically (Fig. 1B).

Control of clock protein stability is a key regulatory step to insure the tightness of circadian rhythms [22], [23]. Moreover, it has been shown that GSK3β-mediated phosphorylation of β-catenin, SRC-3, and Smad3 leads to ubiquitylation followed by proteosomal degradation [24], [25], [26]. Hence, we addressed the question of whether GSK3β affects BMAL1 ubiquitylation. We co-expressed HIS-tagged ubiquitin along with Myc-CLOCK and Flag-Myc-BMAL1 with or without constitutively active (CA, Ser9>Ala mutation) or kinase dead (KD, Lys85>Ala) mutants of HA-tagged GSK3β. Ubiquitylated proteins were pulled down using Ni-Sepharose beads under denaturing conditions and resolved by SDS-PAGE. Upon Western analysis using anti-Flag antibody to specifically detect BMAL1, we observed that co-expression of CA-GSK3β greatly enhanced BMAL1 ubiquitylation, whereas co-expression of KD-GSK3β had no effect on basal BMAL1 ubiquitylation (Fig. 1C). This result confirmed that phosphorylation by GSK3β primes BMAL1 for ubiquitylation. Thus, phosphorylation by GSK3β appears to be an important step in BMAL1 proteosomal degradation, controlling BMAL1 cyclic protein levels.

### Inhibition of GSK3β Activity Enhances BMAL1 Stability

**Figure 2:**
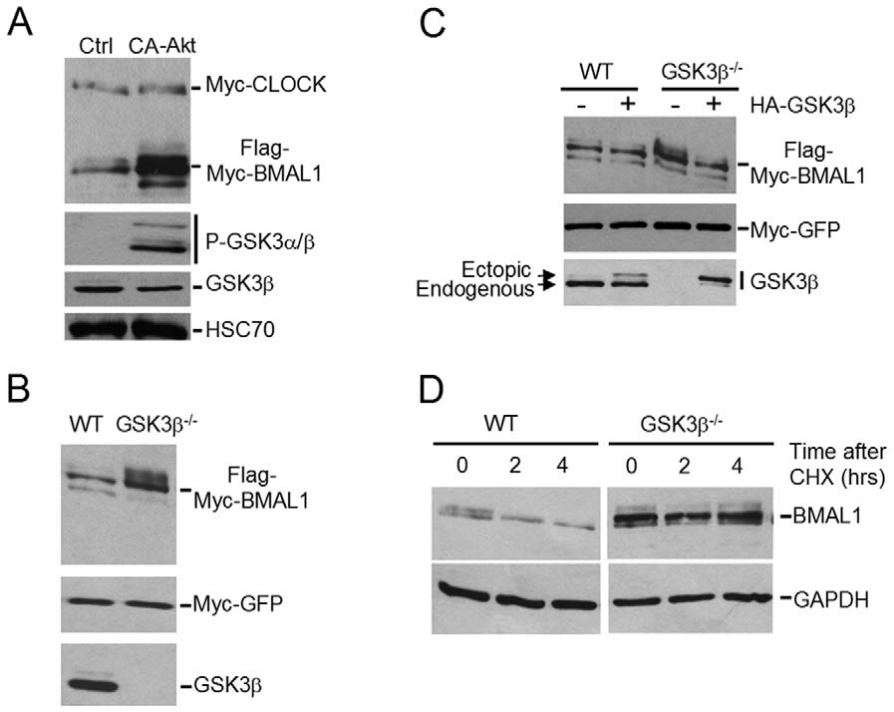
GSK3β regulates BMAL1 stability. (A) HEK 293 cells were co-transfected with plasmids expressing Flag-myc-BMAL1, myc-CLOCK with or without CA-Akt. 48 hours post-transfection nuclear lysates were prepared and resolved by SDS-PAGE. CLOCK and BMAL1 levels were detected by Western analysis using anti-Myc antibody; phospho- and total GSK3β and HSC70 (loading control) were detected by using specific antibodies. (B) WT and GSK3β^−/−^ MEFs were transfected with plasmids expressing Flag-myc-BMAL1, myc-CLOCK and myc-GFP. Nuclear lysates were resolved by SDS-PAGE followed by Western analysis. BMAL1 and GFP levels were detected by anti-Myc antibody. Total GSK3β was detected using a specific antibody. (C) Same as in (B) except that indicated lanes were also transfected with a plasmid expressing HA-tagged GSK3β. (D) WT and GSK3β^−/−^ MEFs were treated with 50 µg/ml Cycloheximide (CHX), total lysates were prepared at indicated times and resolved by SDS-PAGE. Endogenous BMAL1 and GAPDH levels were detected by Western analysis using specific antibodies. Data shown is representative of three independent experiments.

To further prove that phosphorylation by GSK3β affects BMAL1 stability, we sought to inhibit GSK3β activity. Activation of the Akt kinase results in phosphorylation and consequent inactivation of GSK3β [27]. We transiently co-expressed BMAL1 and CLOCK, in presence or absence of a myristoylated form of Akt that turns this kinase in a constitutively active status (CA-Akt). Western analyses confirmed that co-expression of CA-Akt indeed caused an increase in phosphorylation of GSK3β and its consequent inactivation (Fig. 2A). Interestingly, expression levels of BMAL1, but not CLOCK, was highly induced in cells that also expressed CA-Akt (Fig. 2A). These results support the notion that phosphorylation by GSK3β might lead to BMAL1 degradation.

To further substantiate our hypothesis, we ectopically expressed BMAL1 in mouse embryo fibroblasts (MEFs) derived from WT or GSK3β^−/−^ mice. Myc-tagged GFP was co-expressed as a control. We observed that BMAL1 levels were much higher in GSK3β^−/−^ MEFs as compared to WT MEFs (Fig. 2B). GFP levels were similar in both cell lines. These results demonstrate that there is significantly higher accumulation of BMAL1 in the absence of GSK3β. Next, we sought to rescue the GSK3β^−/−^ MEFs by re-introducing GSK3β. BMAL1 was ectopically expressed by transient transfection into WT and GSK3β^−/−^ MEFs, in conjunction or not of a HA-tagged GSK3β. Rescue of GSK3β function in the GSK3β^−/−^ MEFs caused a decrease in BMAL1 protein levels which returned to those in WT MEFs (Fig. 2C).

To determine whether the half-life of the endogenous BMAL1 protein depends on GSK3β, we treated WT and GSK3β^−/−^ MEFs with cycloheximide to block protein synthesis. Importantly, both BMAL1 basal protein levels and its stability are much higher in the absence of GSK3β (Fig. 2D). These results show that phosphorylation of BMAL1 by GSK3β regulates its stability.

### S17 and T21 on BMAL1 Are Targets for GSK3β Mediated Phosphorylation

**Figure 3:**
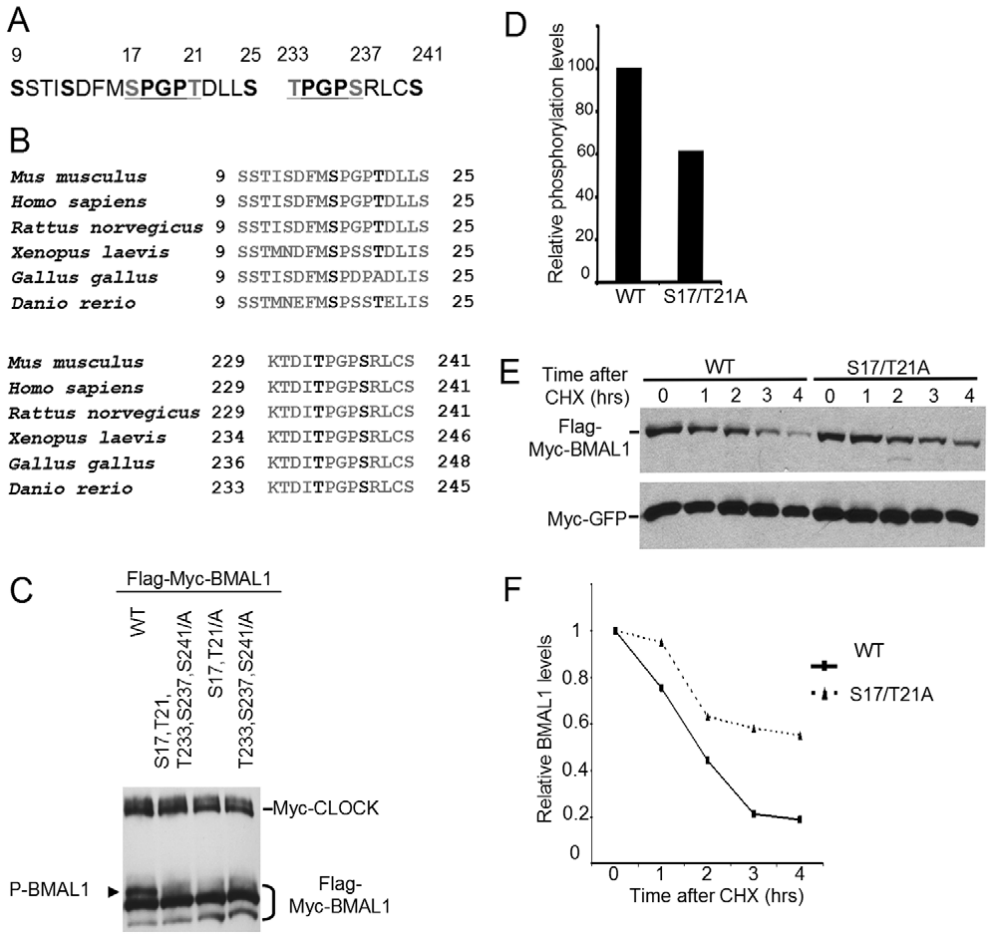
GSK3β phosphorylates Ser 17 and Thr 21 on BMAL1. (A) Amino acid sequence around two putative GSK3β consensus sites on mBMAL1. (B) Sequence alignment of the two putative GSK3β consensus sites on BMAL1 showing conservation across species. (C) HEK 293 were transfected with plasmids expressing myc-CLOCK and WT or mutant Flag-myc-BMAL1. 48 hours post-transfection total lysates were prepared and resolved by SDS-PAGE. BMAL1 and CLOCK levels were detected by anti-Myc antibody. Slower migrating phosphorylated BMAL1 band is indicated by an arrow. (D) Bar graph representing phosphorylation levels of WT and S17,T21/A BMAL1 as analyzed by *In vitro* kinase assay (as in Fig. 1A). Data is representative of three independent experiments. (E) WT or S17,T21/A mutant of Flag-myc-BMAL1, along with myc-GFP were transiently expressed in JEG3 cells and treated with 50 µg/ml CHX. Total lysates were prepared at indicated times and resolved by SDS-PAGE. BMAL1 and GFP levels were detected by Western analysis using anti-myc antibody. Data shown is representative of three independent experiments. (F) Line graph showing quantitation of BMAL1 levels, normalized to GFP levels, from Fig. 3E.

Among the 15 consensus GSK3β sites on BMAL1, we focused our attention on two loci which contained multiple GSK3β consensus sites in tandem. These sites included a region between residues 9 and 25, and another between residues 233 and 241 which are highly conserved in vertebrates (Fig. 3A,B). Importantly, both loci contain a motif which fits a highly stringent consensus for GSK3β mediated phosphorylation [S/TPXXS/T]. Thus, S17 and T21 at the first locus, and T233 and S237 at the second locus, appear to be privileged candidates for phosphorylation by GSK3β. In order to determine if any of these sites are *bona fide* targets for GSK3β, we first analyzed the effect of point mutations at these sites on BMAL1 phosphorylation. We generated a panel of Ser>Ala mutants including: (i) S17 and T21 (S17,T21/A), (ii) T233, S237 and S241 (T233,S237,S241/A), and (iii) S17, T21, T233, S237 and S241 (S17,T21,T233,S237,S241/A). Plasmids expressing either wild type or BMAL1 mutants were co-transfected in HEK 293 cells along with a plasmid expressing Myc-CLOCK and harvested 48 hours post-transfection. Although phosphorylation was reduced in all mutants, mutation of S17 and T21 caused maximal reduction in BMAL1 phosphorylation (Fig. 3C).

To prove that S17 and T21 are directly phosphorylated by GSK3β, we performed an *in vitro* kinase assay with WT and mutant forms of GST-BMAL1. We observed that GSK3β mediated phosphorylation of BMAL1 was reduced when S17 and T21 were mutated (Fig. 3D). This result shows that S17 and T21 are critical sites of GSK3β-mediated phosphorylation, and indicates the presence of yet unrecognized phosphoacceptor sites in the protein. Interestingly, the importance of the S17,T21 sites is validated by the higher, albeit modest, stability of the S17,T21A-BMAL1 mutated protein as compared to the wild type BMAL1 (Fig. 3E,F).

### Oscillation in GSK3β Activity Correlates with BMAL1 Levels

**Figure 4:**
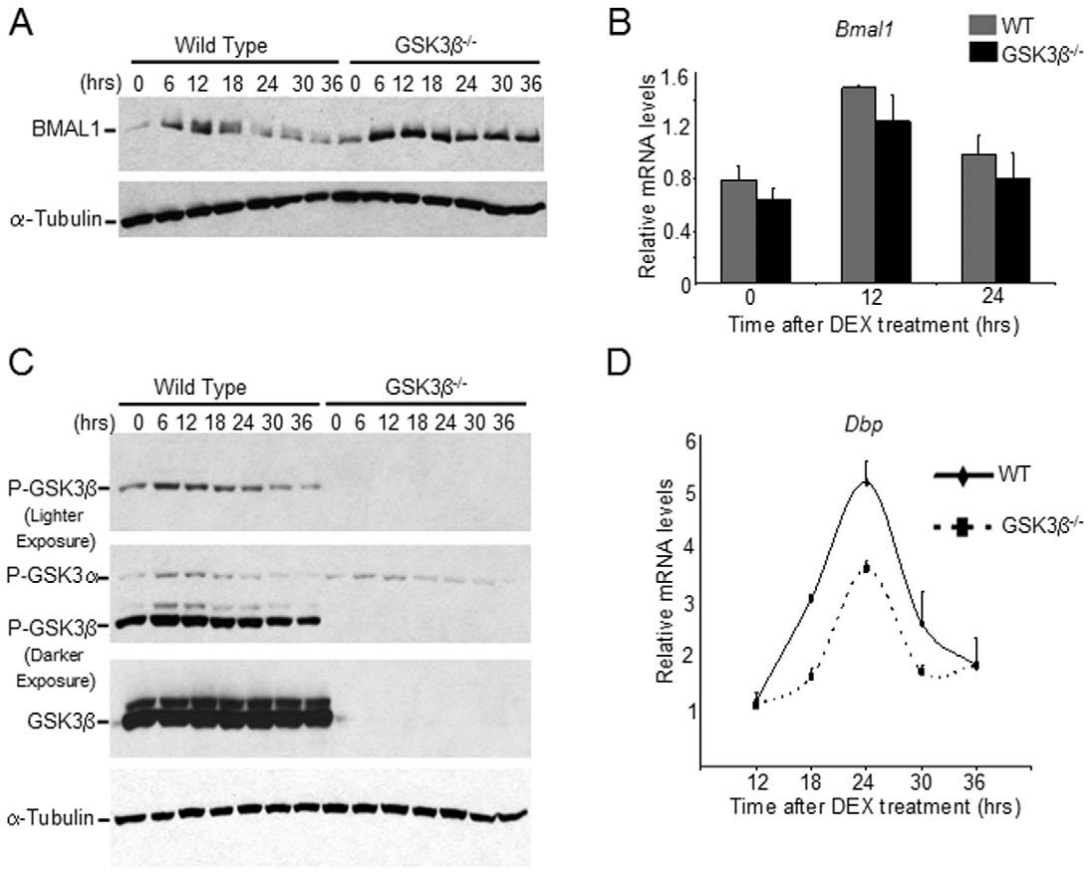
Circadian defects in GSK3β^−/−^ MEFs. WT and GSK3β^−/−^ MEFs were synchronized by 2 hour treatment with 100nM dexamethasone (DEX). (A) Total lysates were prepared at indicated times post synchronization and resolved by SDS-PAGE followed by Western analysis. BMAL1 and α-tubulin levels were detected by specific antibodies. (B) RNA was prepared at indicated times, reverse transcribed, and real-time PCR was performed using primers for *Bmal1* and 18S rRNA. Data is represented as relative levels of *Bmal1* normalized to 18S rRNA. (C) Same as in (A), except phospho-GSK3α/β and total GSK3β levels were detected by specific antibodies. (D) RNA was prepared at indicated times, reverse transcribed, and real-time PCR was performed using primers for *Dbp* and 18S rRNA. Data is represented as relative levels of *Dbp* normalized to 18S rRNA.

Our results show that GSK3β regulates BMAL1 levels. Thus, we questioned whether BMAL1 oscillation would be influenced in the absence of GSK3β. To address this question, we synchronized WT and GSK3β^−/−^ MEFs, and analyzed BMAL1 levels by Western analysis. BMAL1 levels oscillate as expected in WT MEFs, peaking at 6 to 12 hours post-synchronization with lowest levels at 24 hours post-synchronization (Fig. 4A). BMAL1 levels in GSK3β^−/−^ MEFs display only a mild oscillation, with total levels of the protein being much higher at all time points (Fig. 4A). Importantly, the *Bmal1* transcript levels and cyclic expression profile were unchanged in the GSK3β^−/−^ MEFs as compared to WT cells (Fig. 4B). These results indicate that GSK3β is critical for the circadian oscillation of BMAL1 protein. It is, however, important to stress that the residual oscillation of BMAL1 in the GSK3β^−/−^ MEFs indicates the presence of other pathways which may contribute to BMAL1 turnover. One pathway could implicate GSK3α, a kinase structurally similar to GSK3β but not as comprehensively studied.

Next we analyzed whether regulation of GSK3β activity by phosphorylation may oscillate in a circadian manner. Using anti phospho-GSK3β antibodies we reveal that there is more phosphorylation of GSK3β (corresponding to decreased kinase activity) at 6 to 12 hours post synchronization of the MEFs (Fig. 4C). The peak of GSK3β phosphorylation correlates with the increase in BMAL1 protein levels; vice versa, when GSK3β activity is high (24 to 30 hours post synchronization), BMAL1 levels are low (Fig. 4A,C).

As it is not known whether GSK3α activity may be circadian, we analyzed its phosphorylation in parallel with GSK3β (Fig. 4C). GSK3α phosphorylation oscillates with a profile analogous to GSK3β, an event that persisted in GSK3β^−/−^ MEFs. This observation may suggest that GSK3α could partially compensate for the loss of GSK3β in circadian control, although more studies are needed to confirm whether GSK3α plays a role in BMAL1 phosphorylation and turnover.

### GSK3β^−/−^ MEFs Display Dampened Circadian Oscillation of *Dbp* mRNA

The regulation of BMAL1 phosphorylation and stability by GSK3β may impact on the cyclic expression of clock-controlled genes. We thereby compared the expression profile of the *Dbp* gene in WT and GSK3β^−/−^ MEFs synchronized by dexamethasone. Lack of GSK3β resulted in a significant dampening of *Dbp* oscillation as compared to WT MEFs, featuring a conserved period but decreased amplitude (Fig. 4D). These results support the notion that stabilization of BMAL1 protein levels might lead to reduction in its transcription activation ability [28].

### Dopamine Signaling Regulates the Circadian Clock by Controlling BMAL1 Turnover

**Figure 5:**
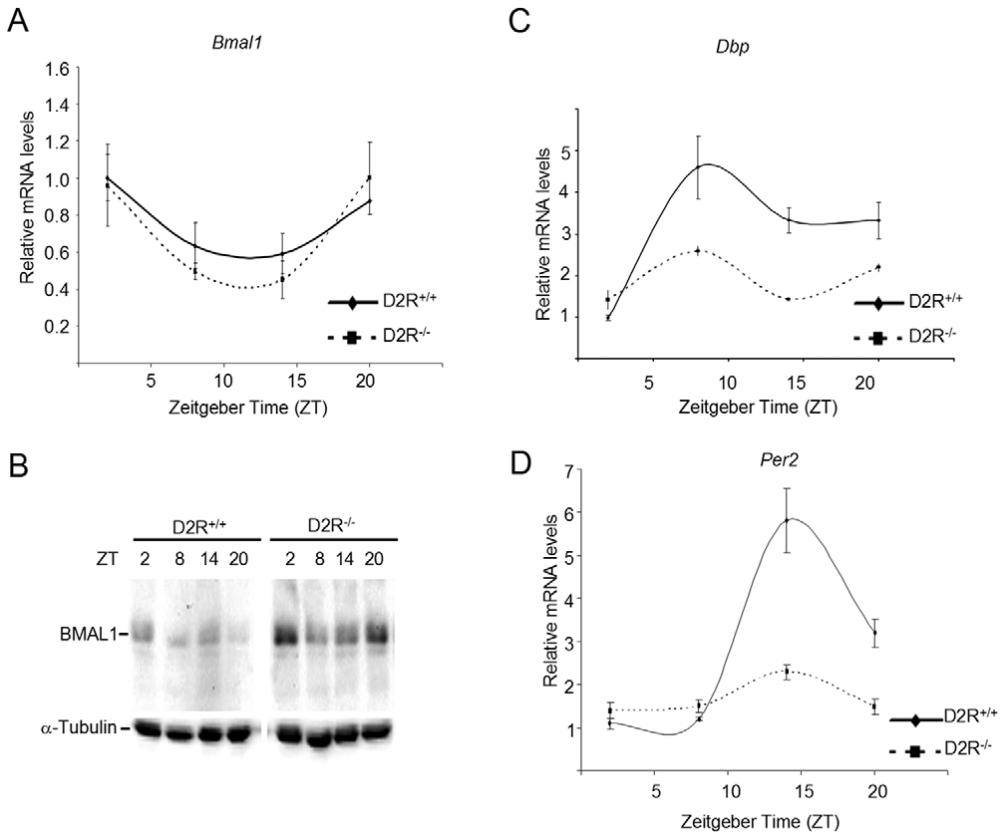
BMAL1 mRNA and protein levels in WT and D2R^−/−^ mice. Mice entrained in 12 hr Light – 12 hr Dark cycles were sacrificed at indicated times and their striatum was dissected out. (A) RNA was prepared at indicated times, reverse transcribed, and real-time PCR was performed using primers for *Bmal1* and 18S rRNA. Data is represented as relative levels of *Bmal1* normalized to 18S rRNA. (B) Total lysates were prepared and resolved by SDS-PAGE. BMAL1 and α-tubulin levels were detected by Western analysis using specific antibodies. (C,D) Same as in (A), except real-time PCR was performed using primers for (C) *Dbp* and (D) *Per2*.

Dopamine signaling via the D2 receptor (D2R) has been demonstrated to modulate the Akt-GSK3β pathway *in vivo* [29]. In addition, we have previously reported that CLOCK∶BMAL1 transcriptional activity is enhanced by activation of D2R-mediated signaling [30]. We thus used the D2R^−/−^ mice [31] as a model to analyze the effect of inactivating the GSK3β pathway *in vivo*. To do so, we isolated the striatum from brains of mice entrained at different times of the circadian cycle (LD 12∶12). While the cyclic expression profile and mRNA levels of *Bmal1* were similar in the striatum from WT and D2R^−/−^ animals (Fig. 5A), the levels of BMAL1 protein were elevated in the D2R^−/−^ mice (Fig. 5B). This result parallels the inactivation of the GSK3β pathway observed in cultured MEFs (Fig. 4). Also, consistent with the expression profile observed in the GSK3β^−/−^ MEFs, the amplitude of the circadian expression of both *Dbp* and *Per2* is significantly decreased in the striatum of D2R^−/−^ mice (Fig. 5C and D). These results confirm that the GSK3β pathway regulates circadian gene expression by controlling BMAL1 protein stability *in vivo*.

## Discussion

BMAL1 is a critical regulator of the circadian clock. Its ablation in the mouse, in addition to causing arrhythmicity [32], leads to premature aging and reduced life-span [33]. BMAL1 is a crucial player in the regulation of metabolism, playing a role in adipogenesis [34] and in the control of glucose and triglycerides levels [35]. Mice with a liver specific deletion of *Bmal1* show normal locomotor activity, but the circadian expression of key metabolic genes, such as *glucose transporter 2* (*glut2*), is abolished. This results in mice being hypoglycemic during the fasting phase of the feeding cycle [36].

The important roles played by BMAL1 parallel multiple levels of regulation of its function. BMAL1 undergoes various PTMs, including phosphorylation, acetylation [7], sumoylation [8], [9] and ubiquitylation [10]. While the functional interactions between these PTMs have not been deciphered, phosphorylation by distinct kinases appears to regulate unique activities [10], [37]. Specifically, phosphorylation by Casein Kinase Iε (CKIε) has been reported to activate BMAL1-mediated transcription [6], while phosphorylation by MAPK inhibits it [4]. Recently we have shown that CK2α-mediated phosphorylation regulates BMAL1 intracellular localization [5]. Our present results indicate that GSK3β activity is circadian and that BMAL1 phosphorylation by this kinase controls its stability.

Our analysis identified S17 and T21 in BMAL1 as major target sites for GSK3β- mediated phosphorylation. While mutation of these residues leads to a reduction of phosphorylation and an increase in BMAL1 stability, we predict the presence of additional GSK3β phosphoacceptor sites on BMAL1. Importantly, multiple phosphorylation events on a clock protein, presumably by multiple kinases appear to be a common mode of regulation. For example, it has been recently shown that the *Neurospora* clock protein FREQUENCY is phosphorylated at 75 residues [38]. The phosphorylation status of several of these residues changes according to the time of the day and regulates period length. In mammals, PER2 has been shown to be phosphorylated at 21 sites, regulating its stability and localization [39]. While a detailed proteomic analysis of BMAL1 phosphorylation is warranted, our results reveal that GSK3β is pivotal in circadian function as it controls the stability of a core clock regulator. Further illustrating the importance of GSK3β in circadian regulation, a recent report demonstrated that GSK3β can also phosphorylate CLOCK and regulate its stability [40]. It would be interesting to explore whether, similarly to what we describe here for dopamine (Fig. 5), some specific pathways known to operate via GSK3β, such as the insulin signaling in liver [15], regulate the circadian clock by altering the phosphorylation state and the stability of BMAL1. The implication of GSK3β in circadian control is significant, especially considering that this kinase has been linked to various diseases such as diabetes, Alzheimer's, cancer and neuropsychiatric disorders. The link between D2R signaling and BMAL1 stability is challenging, as dopamine dysfunctions underlie neurological and psychiatric disorders which are disruptive of the circadian clock. Future studies will determine whether GSK3β-mediated disruption of normal circadian control is causally linked to some of these pathological conditions. In this sense, it is interesting that the activity of GSK3α also oscillates in circadian manner (Fig. 4). Although our data suggests that GSK3α might partially compensate for the loss of GSK3β, further studies are needed to determine whether BMAL1 and other clock components can be phosphorylated by GSK3α.

It could be argued that the effects of GSK3β on BMAL1 are indirect. It has indeed been shown that phosphorylation of Rev-erbα by GSK3β leads to its stabilization [21]. Since Rev-erbα is a negative regulator of *Bmal1* transcription, it can be expected that when GSK3β is inhibited, Rev-erbα will be less stable, and hence, *Bmal1* transcription may be de-repressed. However, our results conclusively demonstrate that the observed stabilization of BMAL1 protein upon GSK3β inhibition is independent of Rev-erbα. The evidence in support of our argument is: (i) BMAL1 protein has a longer half-life in GSK3β^−/−^ MEFs when compared to WT MEFs (Fig. 2D); (ii) BMAL1 accumulation upon GSK3β inhibition is evident not only on endogenous BMAL1, but also on ectopically expressed BMAL1 (Fig. 2). The exogenous BMAL1 is not under control of the *Bmal1* promoter and hence can not be affected by Rev-erbα; and (iii) BMAL1 protein levels are higher in the GSKβ^−/−^ MEFs and D2R^−/−^ mice than in their wild type counterparts, although *Bmal1* circadian mRNA levels are virtually equivalent (Figs. 4 A,B and 5 A,B). Altogether, the present findings unequivocally show that phosphorylation of BMAL1 by GSK3β is a critical step in the control of its stability.

Recent reports have indicated that although circadian locomotor activity persists in mice displaying constitutively higher levels of BMAL1, either due to abolished Rev-erbα expression[41] or by transgenic re-expression in *Bmal1* ^−/−^ mice [42], the period length is rendered shorter. Moreover, robustness in tissue-specific circadian expression of certain clock genes (e.g. *Cry1*, *Dbp*) is compromised in these animals. These observations are in agreement with our data showing that increased BMAL1 protein levels in GSKβ^−/−^ MEFs and D2R^−/−^ mice does not abolish circadian gene expression, but affects its amplitude.

PTMs are often found in specific combinations in order to regulate protein activity [43]. Phosphorylation and ubiquitylation are often found to be coupled. In certain cases, such as β-catenin, IκBα, cyclin E, SREBP1, phosphorylation of a protein generates a “Phosphodegron” which promotes recognition by ubiquitin ligases [43]. Interestingly, in case of some transcription factors, their transcriptional activation domains (TAD) and degrons are overlapping [28]. The “Black Widow” model for transcription activation suggests that certain transcription factors are active only when unstable and their ubiquitylation status positively correlates with their activity. For example, stabilization of VP16 TAD causes its inactivation [44]. The VP16 TAD signals its ubiquitylation through the Met30 ubiquitin ligase. In the absence of Met30, VP16 is more stable but is unable to activate transcription. For BMAL1, it is notable that at specific times of the day when its abundance is low, its activity is high [45]. The low abundance of BMAL1 also coincides with its high phosphorylation. Thus, we speculate that activation by BMAL1 also follows the “Black Widow” model: upon GSK3β mediated phosphorylation and generation of phosphodegrons, ubiquitylation by yet uncharacterized ubiquitin ligases would de-stabilize and thus, activate BMAL1. Identification of the E3 ubiquitin ligase specific for BMAL1, and the sites for ubiquitylation on BMAL1 should be the subject of future studies that would provide invaluable information on the control of circadian rhythms.

## Materials and Methods

### Animals

Generation of D2R-deficient mice has been described [31]. The D2R-null mice and wild type mice used were littermates. Mice housed in individual cages were entrained on a L12∶D12 (12 h light–12 h dark) cycle for two weeks before analyses. Mice were sacrificed at specified circadian times and striata were isolated and homogenized as described (27). All research involving vertebrate animals has been performed under protocol approved by the Institutional Animal Care and Use Committee (IACUC). Animals are monitored on a daily basis by both the lab and University Lab Animal Resources (ULAR) veterinary staff for signs of distress, pain, and/or infection, and are given ad libitum access to food and water. Cages were cleaned on a weekly basis and when visibly soiled to maintain a clean environment. All husbandry procedures and welfare policies are conducted according to the Guide for the Care and Use of Laboratory Animals, set forth by the Institute of Laboratory Animal Resources, Commission on Life Sciences, and National Research Council.

### Reagents

Plasmid expressing a myristoylated form of AKT (constitutively active) was from Millipore (Temecula, CA). Plasmids expressing wild type, constitutively active or kinase dead forms of GSK3β were gifts from Dr. Jim Woodgett (Samuel Lunenfeld Research Institute, Mount Sinai Hospital, Toronto, Canada). Myc-tagged Clock, Flag-Myc- tagged Bmal1, and Myc-tagged GFP plasmids have been described earlier [7]. Antibodies against phospho-GSK3α/β were from Cell Signaling Technology (Danvers, MA); anti-Myc and anti-GAPDH from Millipore (Temecula, CA); anti-flag and anti-tubulin from Sigma (St. Louis, MO); anti-HSC70 from Santa Cruz Biotechnology, Inc (Santa Cruz, CA); and anti-total GSK3β was from BD Biosciences (San Jose, CA). Site directed mutagenesis kit was from Stratagene (La Jolla, CA).

### Cell Culture and Transfection

HEK 293 cells (ATCC, Manassas, VA) were cultured in DMEM supplemented with 10% NCS and antibiotics. JEG3 cells (ATCC, Manassas, VA) were cultured in BME supplemented with 10% FBS and antibiotics. Cells were transfected with indicated plasmids using BioT transfection reagent (Bioland Scientific LLC, Cerritos, CA) according to manufacturer's recommendations. Mouse embryonic fibroblasts (MEFs) were cultured in DMEM supplemented with 10% FBS and antibiotics. Confluent MEFs were synchronized by treatment with 100nM Dexamethasone (Sigma, St. Louis, MO) for 2 hours. Wild type and GSK3β^−/−^ MEFs were generous gifts from Dr. Jim Woodgett.

### Preparation of Total and Nuclear Extracts from Cultured Cells

Cells were washed twice with cold phosphate buffered saline (PBS) and lysed in RIPA buffer (50 mM Tris pH 8.0, 150 mM NaCl, 5 mM EDTA, 15 mM MgCl_2_, 1% NP40, 1× protease inhibitor cocktail (Roche Diagnostics, Indianapolis, IN ), 1mM DTT, 10 mM NaF, 1 mM PMSF). For nuclear extracts, after washing cells with cold PBS, cells were lysed with hypotonic buffer (10 mM HEPES-KOH pH 7.9, 1.5 mM MgCl_2_, 10 mM KCl, 1× protease inhibitor cocktail, 1mM DTT, 10 mM NaF, 1 mM PMSF). Following a brief centrifugation, pellet was resuspended in hypertonic buffer (20 mM HEPES-KOH pH 7.9, 25% glycerol, 420 mM NaCl, 1.5mM MgCl_2_, 0.2 mM EDTA, 1× protease inhibitor cocktail, 1mM DTT, 10 mM NaF, 1 mM PMSF). Supernatants were recovered as nuclear extracts.

### Ubiquitylation Analysis

HEK 293 cells were co-tranfected with plasmids expressing HIS-tagged ubiquitin, Myc-Clock, flag-Myc-Bmal1 and constitutively active (CA) or kinase dead (KD) mutants of HA-tagged GSK3β. 48 hours post-transfection, cells were treated with 10 µM MG132 for 6 hours, and then lysed in denaturing buffer containing 8 M Urea. Ubiquitylated (and thus HIS tagged) proteins were enriched with Ni–sepharose 6 fast flow beads (GE healthcare, Uppsala, Sweden) and ubiquitylated BMAL1 was detected by Western analysis with the antibody against Flag.

### In Vitro Kinase Assay

Glutathione-S-transferase (GST) or GST-BMAL1 proteins were purified from BL21 strain of *Escherichia coli* using glutathione- sepharose beads (GE Healthcare, Uppsala, Sweden). Purified proteins were washed in kinase buffer (20mM Tris-HCl, pH 7.5, 10mM MgCl_2_, 5mM DTT) and then incubated with or without 25 ng recombinant GSK-3β (New England Biolabs, Ipswich, MA) in presence of 200 µM ATP and 5 µCi γ^32^p ATP at 30°C for 30 min. Reaction was terminated by boiling in presence of Laemmli buffer. Samples were resolved by SDS-PAGE, stained by Coomassie blue, destained, dried and then phosphorylated BMAL1 was detected by autoradiography.

### Quantitative Real-Time RT-PCR

Each quantitative real-time RT-PCR was performed using the Chromo4 real time detection system (BIO-RAD). The PCR primers for mBmal1 mRNA, mDbp mRNA, mPer2 mRNA, 18S rRNA are available upon request. For a 20 µl PCR, 50 ng of cDNA template was mixed with the primers to final concentrations of 200 nM and 10 µl of iQ SYBR Green Supermix (BIO-RAD), respectively. The reaction was first incubated at 95°C for 3 min, followed by 40 cycles at 95°C for 30 s and 60°C for 1 min.
